# Supplementary material for: Kinetic characterization of three human DExD/H-box RNA helicases
Source: bioRxiv. 2025 Feb 7:2025.02.07.637080. Preprint. [Version 1] doi: 10.1101/2025.02.07.637080 (PMC11839018; doi:10.1101/2025.02.07.637080)
Supplement: 1 [file NIHPP2025.02.07.637080v1-supplement-1.pdf]

bioRxiv preprint doi: https://doi.org/10.1101/2025.02.07.637080; this version posted February 7, 2025. The copyright holder for this preprint (which was not certified by peer review) is the author/funder, who has granted bioRxiv a license to display the preprint in perpetuity. It is made available under aCC-BY 4.0 International license.

Supporting Figures

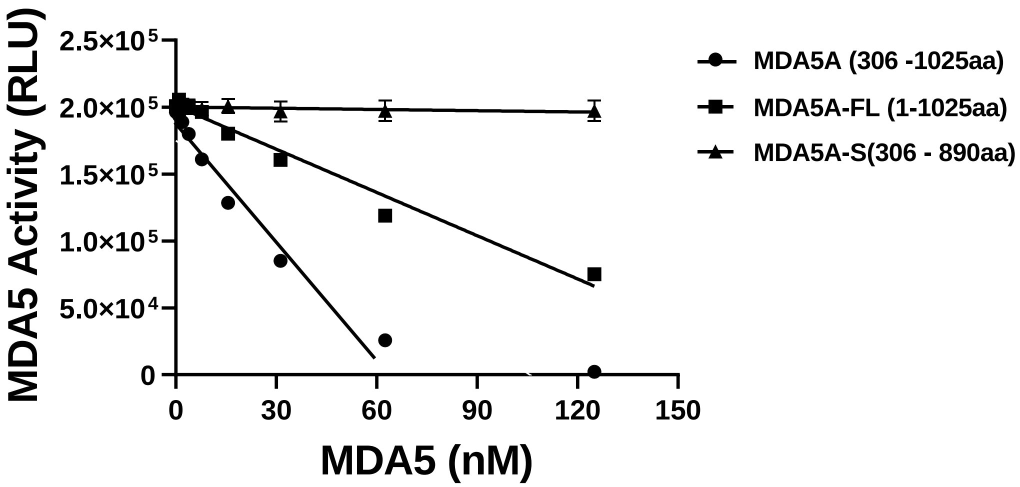

Supplementary Figure S1. ATPase activity of different lengths of MDA5: deleted 1-306aa (●), full-length (■) and truncated 1-306aa and 891-1025aa (▲).

The experiments were conducted in triplicate (n=3).

bioRxiv preprint doi: https://doi.org/10.1101/2025.02.07.637080; this version posted February 7, 2025. The copyright holder for this preprint (which was not certified by peer review) is the author/funder, who has granted bioRxiv a license to display the preprint in perpetuity. It is made available under aCC-BY 4.0 International license.

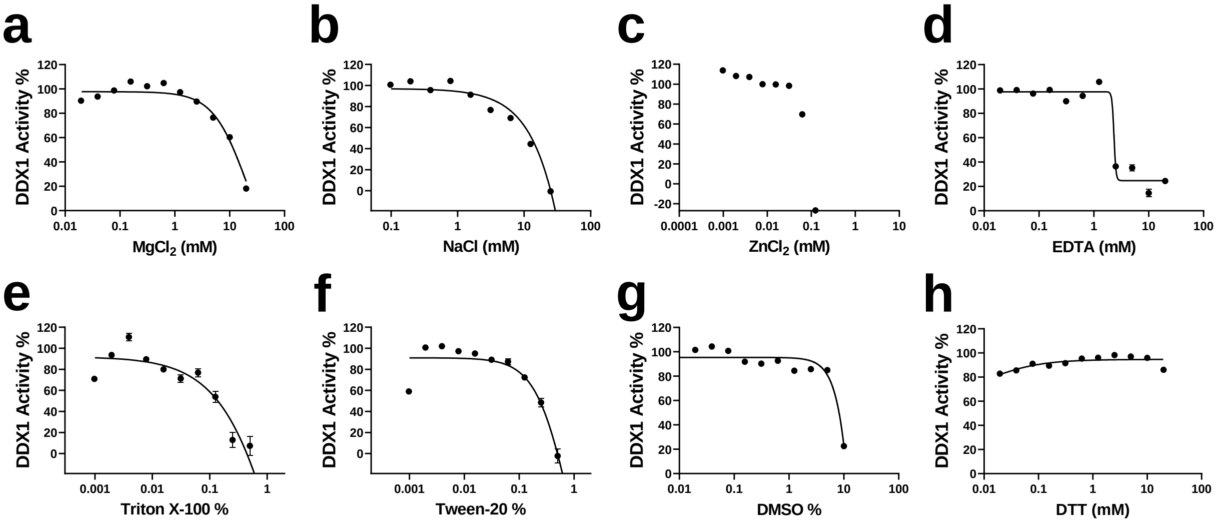

Supplementary Figure S2. **Effect of ionic strength, and buffer additives on DDX1 ATPase activity.** The ATPase activity of DDX1 in the presence of 24-mer RNA was determined as a function of $MgCl_2$ (a), NaCl (b), $ZnCl_2$ (c), EDTA (d), Triton x-100 (e), Tween 20 (f), DMSO (g), and DTT (h) as described under "Methods and Materials". Data points are presented as mean ±S.D. from three experiments.

bioRxiv preprint doi: https://doi.org/10.1101/2025.02.07.637080; this version posted February 7, 2025. The copyright holder for this preprint (which was not certified by peer review) is the author/funder, who has granted bioRxiv a license to display the preprint in perpetuity. It is made available under aCC-BY 4.0 International license.

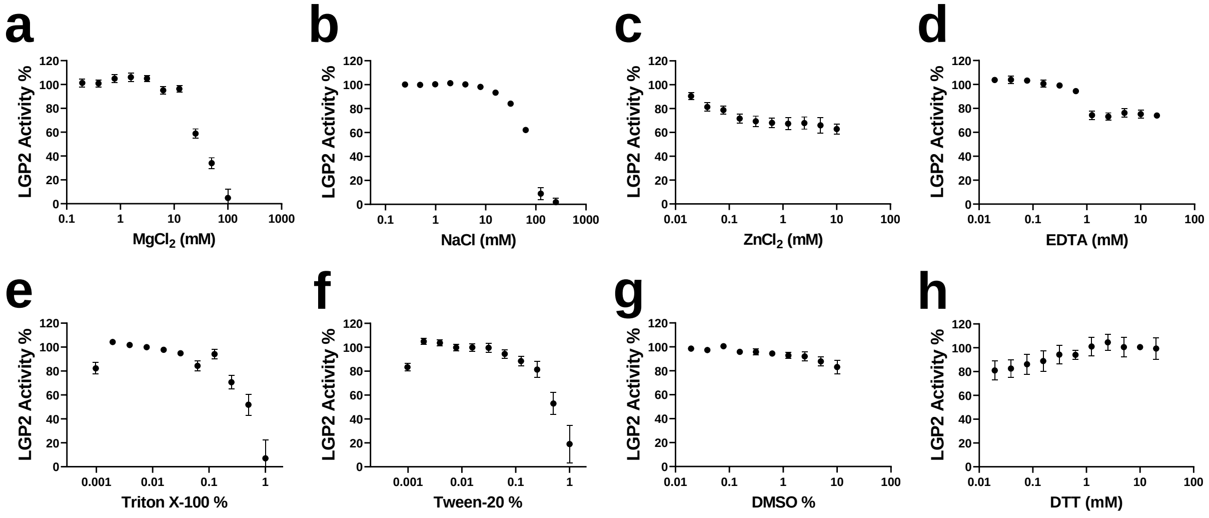

Supplementary Figure S3. **Effect of ionic strength, and buffer additives on LGP2 ATPase activity.** The ATPase activity of LGP2 in the presence of 24 mer RNA was determined as a function of $MgCl_2$ (a), NaCl (b), $ZnCl_2$ (c), EDTA (d), Triton x-100 (e), Tween 20 (f), DMSO (g), and DTT (h) as described under “Methods and Materials”. Data points are presented as mean ±S.D. from three experiments.

bioRxiv preprint doi: https://doi.org/10.1101/2025.02.07.637080; this version posted February 7, 2025. The copyright holder for this preprint (which was not certified by peer review) is the author/funder, who has granted bioRxiv a license to display the preprint in perpetuity. It is made available under aCC-BY 4.0 International license.

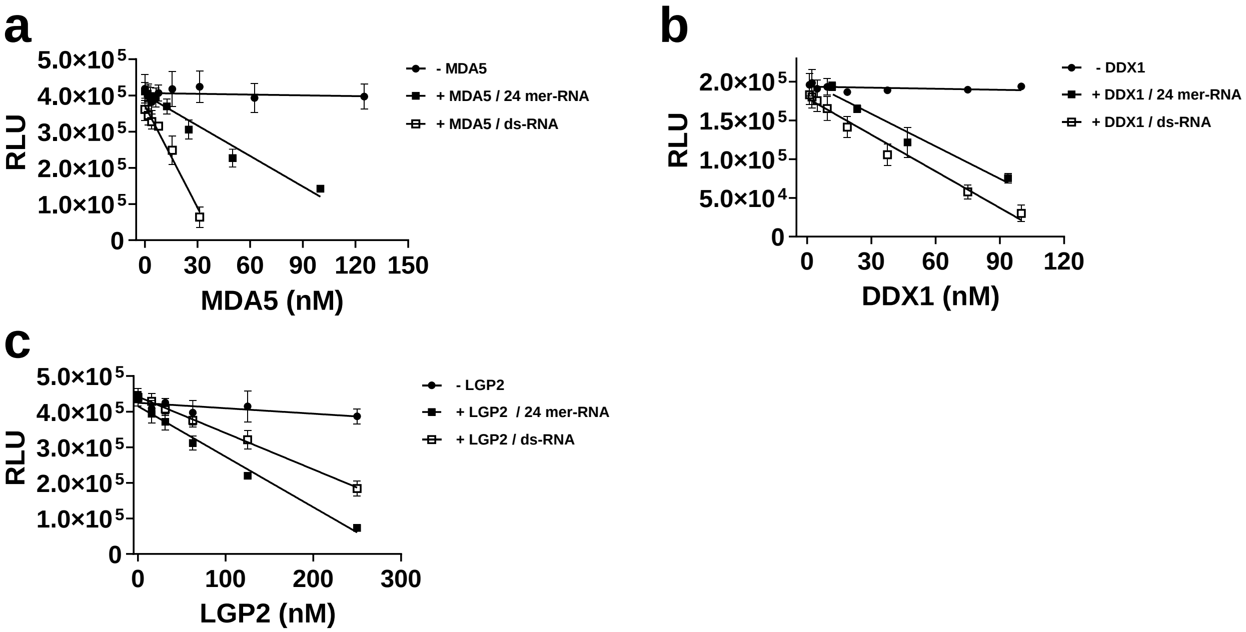

Supplementary Fig. S4. The linearity of ATPase activities for the three helicases was tested at ATP concentrations near their K_m values in the presence of 24mer-RNA. (a) MDA5 activity with varying concentrations or absence of MDA5 (●: 4.0 µM ATP and 24-mer RNA without MDA5; ■: 4.0 µM ATP and 24-mer RNA with different concentrations of MDA5; □: 4.0 µM ATP and 24-mer RNA with different concentrations of MDA5). (b) DDX1 activity with varying concentrations or absence of DDX1 (●: 2.0 µM ATP and 24mer-RNA without DDX1; ■: 2.0 µM ATP and 24-mer RNA with different concentrations of DDX1; □: 2.0 µM ATP and 24-mer RNA with different concentrations of DDX1). (c) LGP2 activity with varying concentrations or absence of LGP2 (●: 4.0 µM ATP and 24-mer RNA without LGP2; ■: 4.0 µM ATP and 24-mer RNA with different concentrations of LGP2; □: 4.0 µM ATP and 24-mer RNA with different concentrations of LGP2). The experiments were performed in triplicate (n=3). RLU: relative light unit of Luminescence.

bioRxiv preprint doi: https://doi.org/10.1101/2025.02.07.637080; this version posted February 7, 2025. The copyright holder for this preprint (which was not certified by peer review) is the author/funder, who has granted bioRxiv a license to display the preprint in perpetuity. It is made available under aCC-BY 4.0 International license.

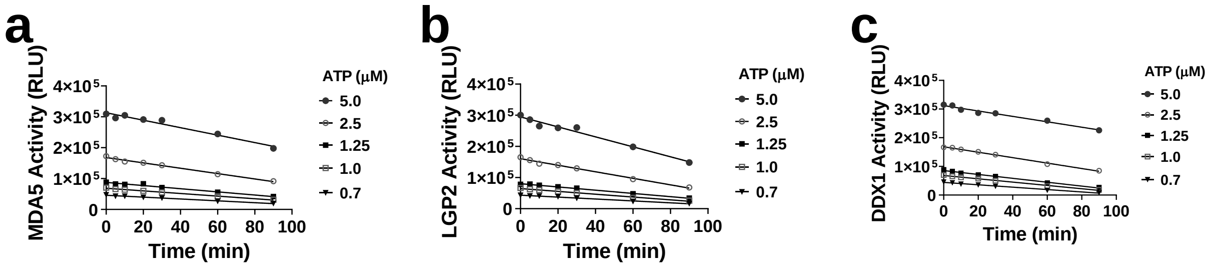

Supplementary Figure S5. Analysis of reaction linearity over time with varying ATP concentrations. The reactions were linear up to 90 min for MDA5 (a), LGP2 (b) and DDX1 (c).

bioRxiv preprint doi: https://doi.org/10.1101/2025.02.07.637080; this version posted February 7, 2025. The copyright holder for this preprint (which was not certified by peer review) is the author/funder, who has granted bioRxiv a license to display the preprint in perpetuity. It is made available under aCC-BY 4.0 International license.

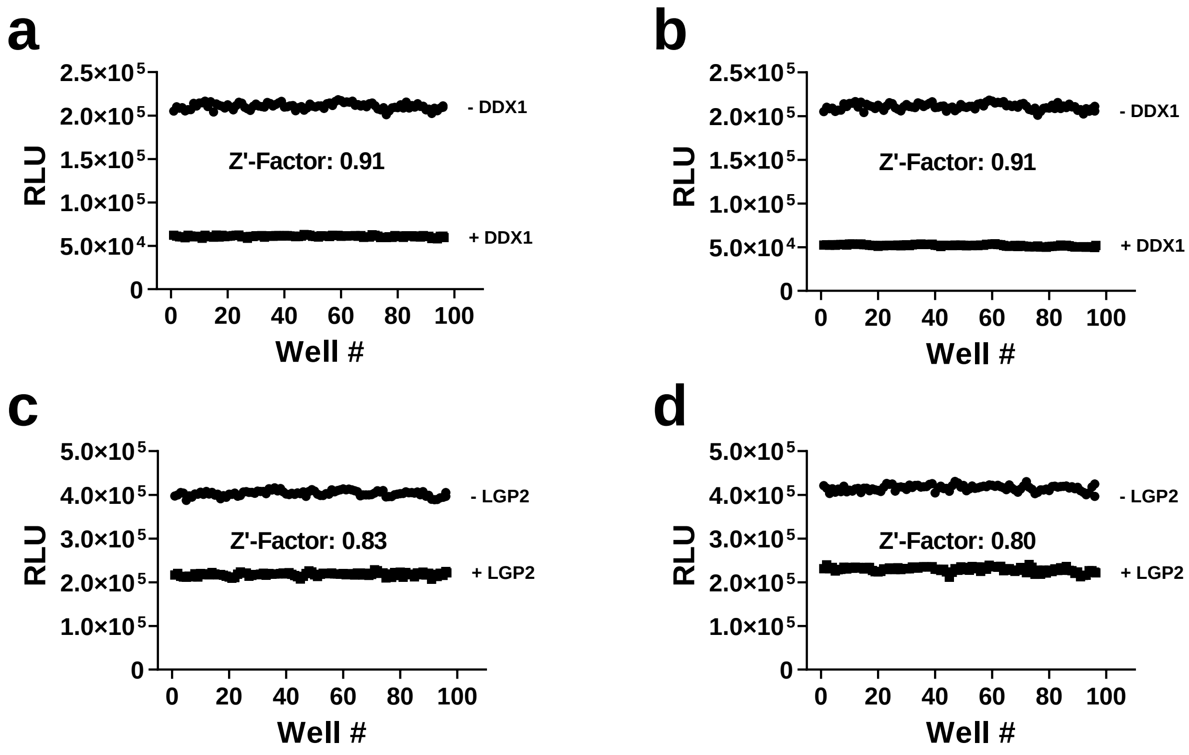

Supplementary Figure S6. Amenability of the assays for high-throughput screening for DDX1, and LGP2

The Z' factors were determined for DDX1 using 2 µM ATP and 100 nM 24-mer RNA (a) and 2 µM ATP with 60nM ds-RNA (b); for LGP2 using 4 µM ATP and 30 nM 24-mer RNA (c) and 4 µM ATP with 30 Nm ds-RNA (d); in the presence (■) or absence (●) of DDX1or LGP2.

bioRxiv preprint doi: https://doi.org/10.1101/2025.02.07.637080; this version posted February 7, 2025. The copyright holder for this preprint (which was not certified by peer review) is the author/funder, who has granted bioRxiv a license to display the preprint in perpetuity. It is made available under aCC-BY 4.0 International license.

```
DDX1      MAAFSEMGVMPEIAQAVEEMDWLLPTDIQAESIPLILGGGDVLMAAETGSGKTGAFSIPV      60
MDA5      ----------------------LQLRPYQMEVAQPALEGKNIIICLPTGSGKTRVAVYIA      38
LGP2      ----------------------MELRSYQWEVIMPALEGKNIIIWLPTGAGKTRAAAYVA      38
                                :     * *     * * ::::   **:*** .    .

DDX1      IQIVYETLKDQQEGKKGKTTIKTGASVLNKWQMNPYDRGSAFAIGSDGLCCQSREVKEWH      120
MDA5      KDH-LDKKKKASEP--GKVIV-----LVNKVLLVEQLFRKEFQ----------PFLKKWY      80
LGP2      KRH-LETVD----G--AKVVV-----LVNRVHLVTQ-HGEEFR----------RMLDGRW      75
               :. .       .*. :     ::*:  :      . *             :.

DDX1      GCRATKGLMKGKHY--YEVSCHDQGLCRVGWSTMQASLDLGTDKFGFGFGGTGKKSHNKQ      178
MDA5      RVIGLSGDTQLKISFPEVVKSCDIIIST--------------------------------      108
LGP2      TVTTLSGDMGPRAGFGHLARCHDLLICT--------------------------------      103
               .*    :      . . *  :.

DDX1      FDNYGEEFTMHDTIGCYLDIDKGHVKFSKNGKDLGLAFEIPPHMKNQALFPACVLKNAEL      238
MDA5      -----------------AQILENSLLNLENGEDAGVQLSDFS----LIIIDECHHTNKEA      147
LGP2      -----------------AELLQMALTSPE--EEEHVELTVFS----LIVVDECHHTHKDT      140
                            :: :  :   :  ::  : :          :.  *  .: :
                                                      DExH-box
DDX1      KFNFG---------------EEEFKFPPKDGFVALSKAP---------------------      262
MDA5      VYNNIMRHYLMQKLKNNRLKKENKPVIPLPQILGLTASPGVGGATKQAKAEEHILKLCAN      207
LGP2      VYNVIMSQYLELKLQ---------RAQPLPQVLGLTASPGTGGASKLDGAINHVLQLCAN      191
           :*                        *   .:.*: :*

DDX1      -DGYIVKSQHSGNAQVTQTKFLPNAPKALIVEPSRELAE-----------QTLNNIKQFK      310
MDA5      LDAFTIKTVKENLDQLKN----------QIQEPCKKFAIADATREDPFKEKLLEIMTRIQ      257
LGP2      LDTWCIMSPQNCCPQLQE----------HSQQPCKQYNLCHRRSQDPFGDLLKKLMDQIH      241
           * : : :.   *: :             :*.::                 : : :::

DDX1      KYIDNPKLRELLIIGGVAARDQLSVLENGVDIVVGTPGRLDDLVSTGKLNLSQVRFLVLD      370
MDA5      TYCQMSPM----------------------------------------------------      265
LGP2      DHLEMPELS---------------------------------------------------      250
           : :   :
         DEAD-box
DDX1      EADGLLSQGYSDFINRMHNQIPQVTSDGKRLQVIVCSATLHSFDVK-------KLSEKIM      423
MDA5      --SDFGTQPYEQWAIQMEKKAA---KEGNR-KERVCAEHLRKYNEALQINDTIRMIDAYT      319
LGP2      --RKFGTQMYEQQVVKLSEAAA---LAGLQ-EQRVYALHLRRYNDALLIHDTVRAVDALA      304
              : :* *.:   :: :        * : :  * :  *: ::         :  :

DDX1      HFPTWV---------------DLKGEDSVPDTV---HHVVVPVN-PKTDRLWERLGKSHI      464
MDA5      HLETFYNEEKDKKFAVIEDDSDEGGDDEYCDGDEDEDDLKKPLKLDETDRFLMT---LFF      376
LGP2      ALQDFYHREHVTKTQILCAE-----------------------------RRLLA---LFD      332
           :  :                                            *        .

DDX1      RTDDVHAKDNTRPGANSPEMWSEAIKILKGEYAVRAIKEHKMDQAIIFCRTKIDCDNLEQ      524
MDA5      ENNKMLKRLAENPEYE-----NEKLTKLRNTIMEQYTRTEESARGIIFTKTRQSAYALSQ      431
LGP2      DRKNELAHLATH-GPE-----NPKLEMLE-KILQRQFSSSNSPRGIIFTRTRQSAHSLLL      385
            ..   :   .   :     .  :  *.     :     :  :.*** :*: ..  *

DDX1      YFIQQGGGPDK--KGHQFSCVCL---HGDRKPHERKQNLERFKKGDVRFLICTDVAARGI      579
MDA5      WITENEKFAEVGVKAHHLIGAGHSSEFKPMTQNEQKEVISKFRTGKINLLIATTVAEEGL      491
LGP2      WLQQQQGLQTVDIRAQLLIGAGNSSQSTHMTQRDQQEVIQKFQDGTLNLLVATSVAEEGL      445
          :: ::        :.: :  .         . .:::: :.:*: * :.:*:.* ** .*:
```

bioRxiv preprint doi: https://doi.org/10.1101/2025.02.07.637080; this version posted February 7, 2025. The copyright holder for this preprint (which was not certified by peer review) is the author/funder, who has granted bioRxiv a license to display the preprint in perpetuity. It is made available under aCC-BY 4.0 International license.

```
DDX1      DIHGVPYVINVTLPDEKQNYVHRIGRVGRAERMGLAISLVATEKEKVWYHVCSSRGKGCY      639
MDA5      DIKECNIVIRYGLVTNEIAMVQARGRARADES---T------------YVLVAHSGSGVI      536
LGP2      DIPHCNVVVRYGLLTNEISMVQARGRARADQS---V------------YAFVATEGSREL      490
          **     *:.  *  ::   *:  **.   :    .            * . :  *.

DDX1      NTRLKEDGGCTIWYNEMQLLSEIEEH-LNCTISQV----------------EPDIKVPVD      682
MDA5      ERETVNDFREKMMYKAIHCVQNMKPEEYAHKILELQMQSIMEKKMKTKRNIAKHYKNNPS      596
LGP2      KRELINEALETLMEQAVAAVQKMDQAEYQAKIRDLQQAALTKRAAQAAQRENQRQQFPVE      550
          : .  ::   .:  : :  :.::.      .* ::                    :   .

DDX1      EFDGKVTYGQKRAAGGGSYKG-----HVDILAPTVQELAALEKEAQ-TSFLHLGYL----      732
MDA5      LITF-LCKNCSVLACSGEDIHVIEKMHHVNMTPEFKELYIVRENKT-LQKKCADYQINGE      654
LGP2      HVQL-LCINCMVAVGHGSDLRKVEGTHHVNVNPNFSNYYNVSRDPVVINKVFKDWKPGGV      609
           .   :  .    .  *.        *   : * ..:   : .:    .    .:

DDX1      ---------------------PNQLFRTF-------------------------------      740
MDA5      IIC-KCGQAWGTMMVHKGLDLPCLKIRNFVVVFKNNSTKKQYKKWVELPITFPNLDYSEC      713
LGP2      ISCRNCGEVWGLQMIYKSVKLPVLKVRSMLLE--TPQGRIQAKKWSRVPFSVPDFDFLQH      667
                               *   .*.:

DDX1      -----------    740
MDA5      CLFSDED----    720
LGP2      CAENLSDLSLD    678
```

Supplementary Figure S7. Amino acid sequence alignment of three human helicases. All amino acid sequences were obtained from the Uniprot database and were aligned using Clustal Omega program.
